# The Methods for Estimating State of Charge in Lithium-Ion Batteries

**DOI:** 10.3390/ma19061267

**Published:** 2026-03-23

**Authors:** Peilin Xu, Ruyan Zhou

**Affiliations:** Collage of Engineering Science and Technology, Shanghai Ocean University, Shanghai 201306, China; 15638516250@163.com

**Keywords:** lithium-ion battery, SOC estimation, open-circuit voltage method, ampere-hour integration method

## Abstract

It is of great significance in real time to accurately monitor the internal state parameters of lithium-ion batteries toy ensure the safety, reliability and lasting efficiency of battery energy storage systems. The battery management system can monitor the working state, prevent overcharge or overdischarge, and make the working process more safe and reliable. The state of charge (SOC) is one of the most important indicators to monitor a working battery, and its accurate estimation is the most important work at present. SOC cannot be measured directly, so the state estimation problem of batteries is transformed into a state estimation problem of time-varying nonlinear systems, the core of which is how to obtain a more accurate and reasonable state estimation value in real time. This paper introduces the definition of battery charge state, summarizes common estimation methods and disadvantages of the ampere-hour integration method and open-circuit voltage method, and finally points out the future development direction of battery charge state estimation methods.

## 1. Introduction

The lithium-ion battery (LIB) has become the main power supply equipment in many fields for its high energy density, electrochemical stability, long charge and discharge life, and environmental friendliness, but it also poses new challenges to its life, safety and other aspects. The battery management system (BMS) is the link between the battery and the user, and it can monitor the state of the battery and prevent the battery from overcharge, overdischarge and other phenomena to ensure the efficient, safe and reliable operation of the battery [1,2]. The main functions of the BMS are monitoring the health state of the battery, monitoring the SOC of the battery, and thermal management. This paper introduces an estimation method of SOC, one of the hot spots. SOC can not only describe the remaining power and remaining working time of a battery, reducing the damage caused by overcharge or overdischarge, but also make the user better understand the state of the battery and improve the energy utilization rate of the battery [3,4]. However, if the battery is not managed properly, there will be serious consequences. For example, in Fengtai County, Huainan, Anhui province, a car suddenly spontaneously ignited, and fire quickly ignited the car body. According to the investigation, the cause of the fire was the aging electrical circuit. On the Beijing–Hong Kong–Macao Expressway in Xiaogan, a semi-truck carrying seven cars suddenly caught fire. The cause of the fire was the aging short circuit of the front line of the semi-trailer truck. In 2016, the Samsung Galaxy Note 7 phone was released. When it was just over a month old, more than 30 explosions and fires caused by battery defects occurred around the world. Samsung later announced that the cause of the combustion was that the battery of the Galaxy Note 7, including the negative plate, was compressed; there were welding machine burrs; and part of the cell did not use insulation tape.

Several factors are closely related to the SOC, such as residual capacity, internal resistance, open circuit voltage (OCV), and output power. SOC cannot be measured directly, and it can only be estimated by assessing measurable parameters such as battery voltage, current, and temperature. Different operating conditions and aging conditions can also affect the prediction accuracy of SOC. In this respect, a typical performance indicator for the quantitative evaluation and comparison of various SOC estimation methods is the mean average error (MAE), which refers to the actual value and the mean difference between the estimated value and the total number of samples, and the speed of estimation [5,6]. Battery management systems are widely used in every link of industrial production. Wang et al. accurately estimated the SOC of LIBs and carried out health management of LIBs according to the operating conditions of the ship, so that the LIBs could work within the safe range and ensure the safe operation of the ship [7]. Song et al. analyzed the relationship between the SOC of the main battery and the working current, working environment and temperature for the LIB of a manned submersible at different depths, and they found that the low-temperature environment of the deep sea and different working currents would have a significant impact on the SOC of the LIB [8]. Zhang et al. considered the special operating environment of mining energy storage batteries, improved the ampere-hour integration method of estimated SOC, and improved the estimation accuracy of SOC [9]. Xiong et al. used the improved algorithm to estimate LIB SOC at different temperatures, effectively solving the problem that a special robot’s LIB SOC cannot be accurately estimated [10]. Thus, the accurate estimation of SOC is of great significance for the application of industrial production and life.

The commonly used SOC estimation methods include the OCV method, ampere-hour integral method, discharge method, Kalman filter method, and neural network method. Although many review articles have addressed various SOC estimation techniques, control-oriented and targeted analyses have not yet been performed. Furthermore, discussion and comparison of various methods was the main focus of previous reviews of SOC assessment. The classification of SOC estimation techniques developed from the optimization, combined, and joint state estimation (co-estimation) methods has not been widely discussed. Based on the above analysis, this paper compares various estimation methods, points out the problems of each method, and gives the future development direction of SOC estimation methods.

## 2. Definition of SOC

(1) The perspective of capacity

SOC is defined as the ratio of the remaining available capacity of the battery to the rated capacity of the battery after being completely static. Accordingly, SOC is commonly used as a percentage. SOC = 0% means that the battery is fully discharged; SOC = 100% means that the battery is fully charged. It can be calculated by the following formula:

(1) $$\mathrm{SOC}=\frac{Q}{Q_{N}}\times 100\%$$

where *Q* is the remaining available capacity, and Q_N_ is the rated capacity of the battery [11].

(2) The perspective of energy

The SOC is defined as the ratio of the remaining energy of the battery to the total available energy:

(2) $$\mathrm{SOC}=\frac{E}{E_{N}}\times 100\%$$

E is the remaining energy of the battery, and E_N_ is the total available energy of the battery [12,13].

## 3. Traditional SOC Estimation Method

The SOC estimation method for LIBs is the most critical problem in the BMS. However, because SOC cannot be directly measured and is easily affected by temperature, charge and discharge rate, aging degree, and other factors, the accurate estimation of SOC has always been the focus and difficulty in research. Traditional SOC estimation methods are experiment-based techniques, which rely on battery parameters such as current, voltage, impedance, and the nominal capacity of the battery. Therefore, the traditional estimation methods can be divided into two categories. One is the direct measurement estimation method, including the OCV method, electro-momentum method, internal resistance method, and AC impedance method. The other is the counting method, or the ampere-hour integration method.

### 3.1. Open-Circuit Voltage Method

**Figure 1:**
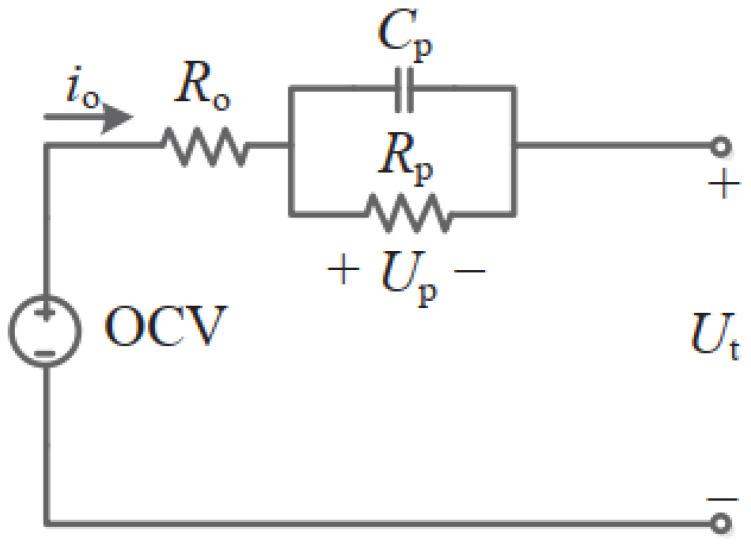
First-order Thevenin equivalent circuit diagram.

**Figure 2:**
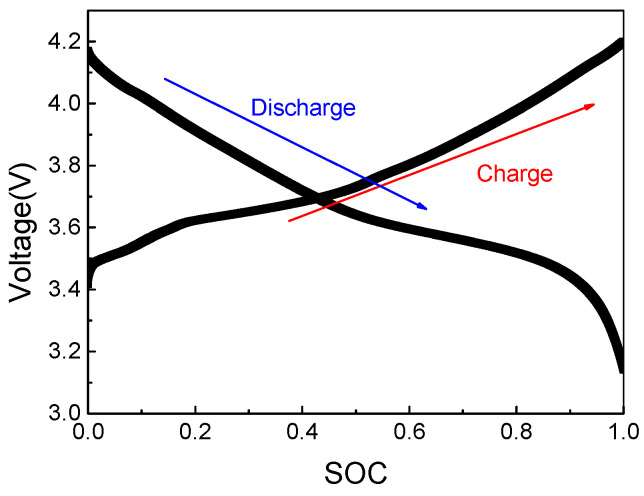
The relationship of OCV and SOC.

OCV refers to the potential difference between the positive and negative electrodes of the battery when no current passes in the circuit [14,15,16]. After the battery has been standing for a long time, the OCV can reflect the current SOC condition of the battery, which is represented by the OCV–SOC curve. After measuring the OCV of the battery, the OCV–SOC relationship can be represented by OCV = *f*(SOC). The OCV method uses the battery characteristic parameters and measurement values, as shown in Figure 1. We measured the relationship between OCV and SOC (Figure 2), which agreed with the literature [14].

The OCV method is particularly widely used. For example, Lin et al. proposed two open-circuit voltage test methods: incremental OCV and low-current OCV. In different temperature and stages, the effects of two open-circuit voltage methods on the SOC estimation of a LiNiMnCoO_2_ battery and a LiFePO_4_ battery are studied, and the results show that incremental OCV is closer to the true value than the results of the low-current OCV test [15]. Because the battery will demonstrate an aging phenomenon after a long time, the OCV–SOC relationship cannot be used for the whole cycle of SOC estimation. OCV needs to be regularly tested to correct the OCV–SOC relationship. Xiong et al. proposed using the filter from the existing current-voltage measurement in the OCV–SOC relationship, and in constant current conditions and dynamic conditions, in order to obtain a result closer to the actual value of the SOC [16]. Pankaj et al. proposed an estimation scheme to track the leakage current and OCV–SOC in real time, which led to the accurate estimation of SOC. The adopted OCV-based equivalent circuit model can effectively obtain the leakage effect of ultracapacitors. The estimation of battery SOC will be affected by various factors [17]. If these factors are not considered, it will lead to great estimation error. By studying the relationship between SOC and OCV in lithium-ion batteries at different times, different temperatures and different SOC states, the accuracy of the OCV method in the estimation of SOC can be improved [14]. The root mean square error, the length of the data segments, and the OCV overlapping range can affect the error of the initial SOC [18].

**Figure 3:**
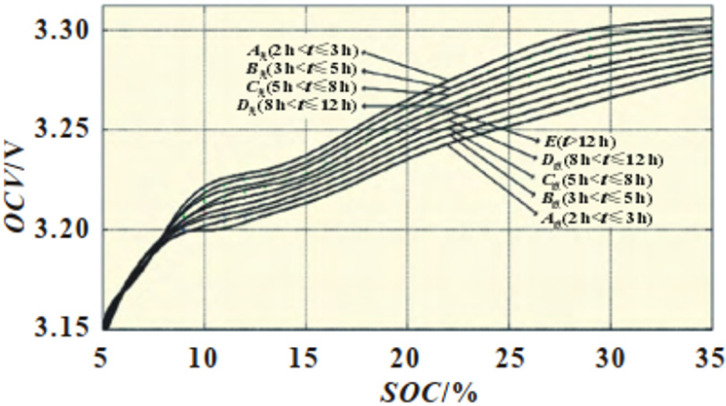
OCV–SOC curve after standing for 2 h.

**Figure 4:**
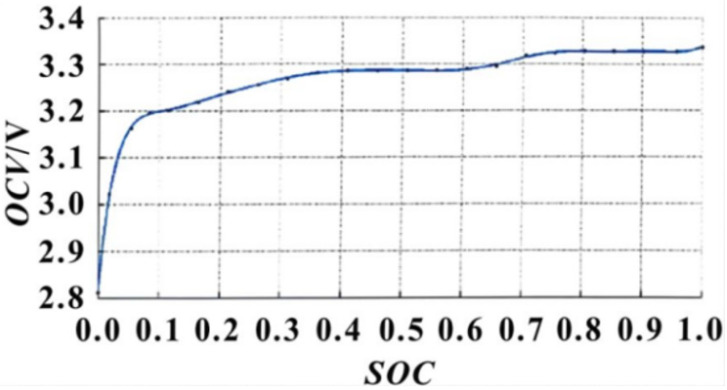
OCV–SOC curve cluster of lithium-ion batteries at different standing times.

It is simple and convenient to estimate SOC by the OCV method. The SOC can be estimated directly by checking the relationship table of OCV–SOC. However, the OCV method also has some disadvantages, and the significant shortcomings are mainly manifested in three aspects. First, after the battery is charged and discharged, it must stand for long enough to achieve complete static state. The resting time m has a relationship with the SOC of the battery, when the standing time t > m. The battery is in a fully stationary state. The OCV at this time can be used to estimate the SOC. When t < m, the battery does not reach the full standing position, and the OCV at this time could not be used to estimate the SOC. Figure 3 and Figure 4 show the resting time m and SOC [19]. Depending on the resting time, we can use the OCV–SOC curve cluster to find the corresponding curve and accurately correct the SOC.

Second, there is a lag effect. Due to the existence of the lag effect, the actual OCV and SOC are not in a one-to-one corresponding relationship, the OCV–SOC curve in the charging state is not the same as the OCV–SOC curve in the discharge state, and a different OCV may be obtained in the same SOC [20]. Third, plateau regions exist in the OCV–SOC curve. Due to the existence of a platform effect, if the OCV segmentation is not sufficient on the platform, a small OCV change will lead to a large change in SOC, and the estimation method will cause a large SOC error [18,21].

The simplicity and convenience of the open-circuit voltage method give this method a large space for use, but due to its several significant and inevitable shortcomings, the open-circuit voltage method is not applicable in some situations, requiring real-time estimation.

### 3.2. Ampere-Hour Integral Method

The ampere-hour integral method only focuses on the external characteristics of the battery. By measuring the charging and discharging time of the battery and the size of the current, the battery can estimate the remaining power of the battery at any moment. The principal formula of the ampere-hour integral method [22] is shown in Equation (3):

(3) $$\mathrm{SOC}=SOC_{0}-\frac{1}{C}\int_{0}^{t}Idt$$

$SOC_{0}$ is the initial power of the battery, C is the rated capacity of the battery, *I* is the charge and discharge current of the battery, *I* < 0 when the battery is charged, and *I* > 0 when the battery is discharged.

The ampere-hour integral method is small, simple, and convenient, and the most commonly used. However, because the ampere-hour integral method is an open-loop algorithm, the error will accumulate over time, and the ampere-hour integral method is susceptible to various factors, such as initial power, temperature, and cycle times. The ampere-hour integral method must be corrected to obtain accurate SOC.

(1) Correction of the initial electric quantity of $SOC_{0}$

**Figure 5:**
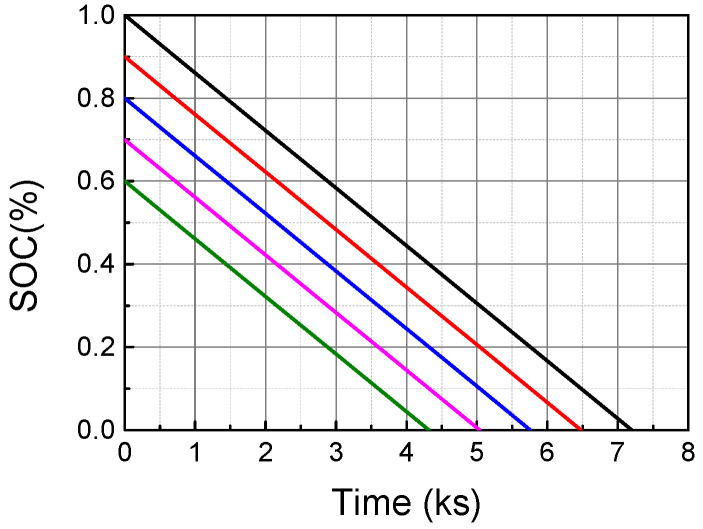
The SOC at the different initial states.

The constant current discharge experiment was performed on the cells at different initial charges. As can be seen from the Figure 5, the estimation results of SOC vary greatly at different initial power levels, so the initial power levels of the battery should be corrected to make the estimation of SOC more accurate.

From the current research progress, the open-circuit voltage method is often used to correct the initial electricity quantity. Because the shortcoming of OCV method is that the battery needs to be left standing for a long time to be completely still, the OCV method is not suitable for battery real-time estimation. However, at the beginning of the battery charge and discharge, the battery has been resting in a completely static state for a long time. The OCV method can be used to estimate the SOC of the battery. Therefore, by measuring the OCV of the initial battery, the initial power can be obtained by checking the table [21,23].

**Figure 6:**
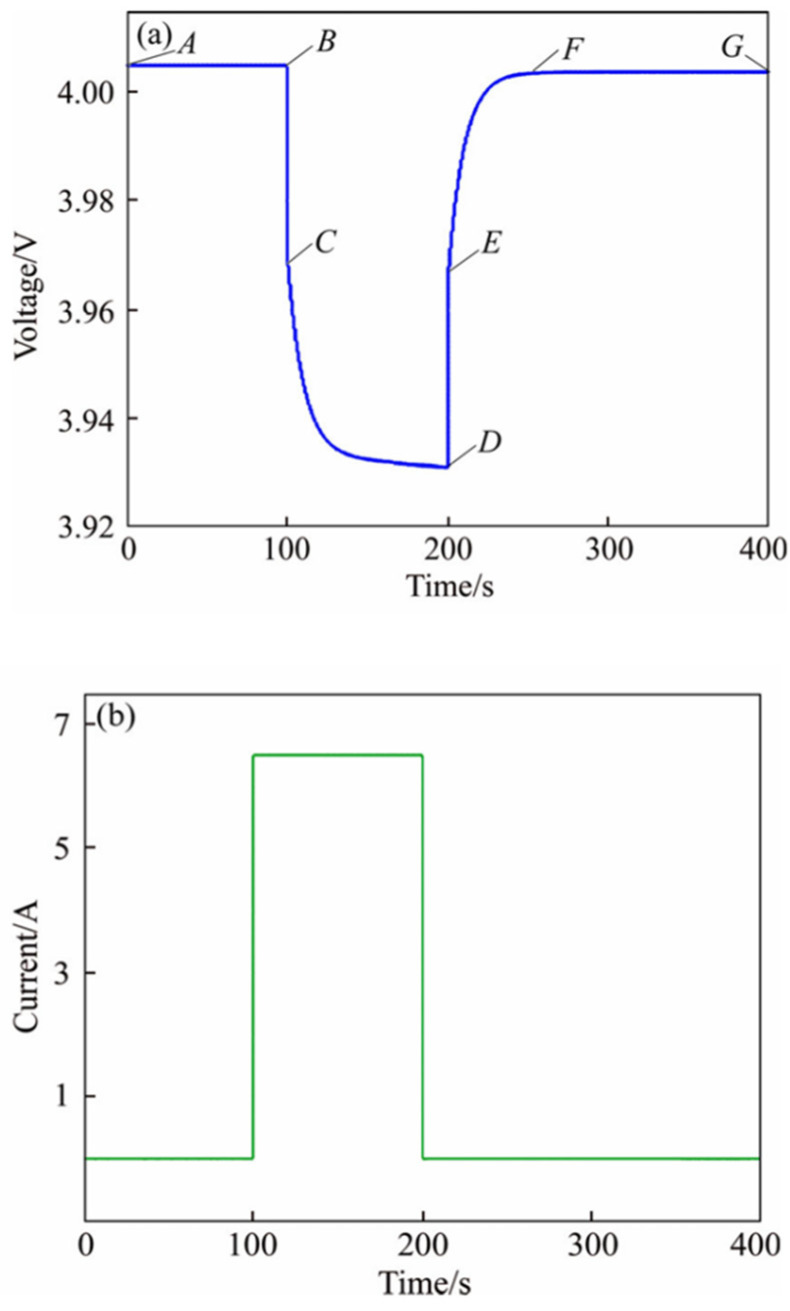
Pulse discharge test curve: (**a**) terminal voltage of battery; (**b**) current of battery. A-B is low current condition, B-C-D is high current condition, D-E-F is current reduction process and F-G is low current condition.

However, it is difficult to estimate the SOC of a battery when the battery is not in the beginning or end stage. If the resting time is greater than x, it means that the battery is completely static, and the OCV method can be used to estimate the battery $SOC_{0}$. If the resting time is lower than x, the battery is not completely set, the $SOC_{0}$ of the battery cannot be estimated by the OCV method, and the battery $SOC_{0}$ obtained the last time is used. The variable x refers to the time it takes for the increase rate of the battery voltage to drop below 1 mV/min as it approaches the end voltage. The OCV relationship between the time and voltage is shown in Figure 6 [24].

When the battery is in the plateau period, the influence of the voltage detection error on the calibration is significant, and the difference in voltage change is still satisfied:

(4) $$V\left(\mathrm{SOC}+n\%\right)-V\left(\mathrm{SOC}\right)>2k$$

where k is the voltage detection error, and the estimated error of the SOC is within n%. The battery detection error of the battery management system is ±5 mV, so Formula (4) is changed to:

(5) $$V\left(\mathrm{SOC}+n\%\right)-V\left(\mathrm{SOC}\right)>10\;\mathrm{mV}$$

**Figure 7:**
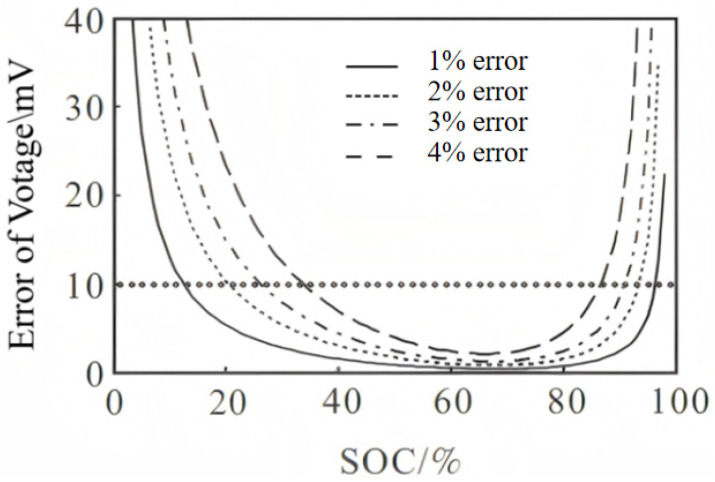
Relationship between battery SOC and voltage difference.

Figure 7 shows the relationship between the SOC and the voltage error. According to the figure, when the SOC is in the lower region S1 and the higher region S2, the calibration is suitable [25]. It can be seen that the error is big when the SOC is low or high.

(2) Correction of temperature factors

The temperature of the battery will affect the conductivity of the electrolyte and the activity of the electrode material. Generally, when the battery temperature increases, the activity of the electrode material increases, and the electrolyte conductivity and the internal resistance of migration decrease. When the battery temperature decreases, the activity of the electrode material decreases, and the energy utilization of the battery decreases too [26,27]. Therefore, the traditional ampere-hour integral method may cause an error in SOC estimation due to the influence of temperature factors. Based on the above analysis, the temperature should be corrected.

**Figure 8:**
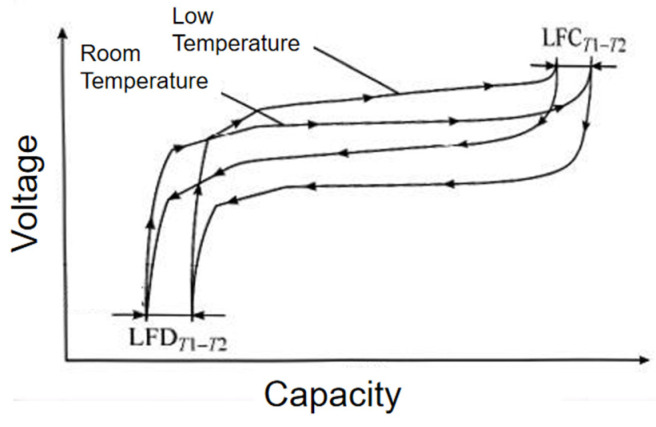
Conversion process of the SOC at different temperatures.

Although the same battery has the same amount of power, the SOC is different when the temperature is different, so the SOC at different temperatures should be converted [28]. Figure 8 shows the conversion process of SOC at different temperatures, and the loss of full discharge capacity is ${\mathrm{LFD}}_{T1-T2}$. The following equation demonstrates this:

(6) $$\mathrm{SOC}\left(t\right)={\mathrm{SOC}}_{T}\left(t-1\right)+\int_{0}^{1}\frac{I}{C_{T}}\mathrm{dt}$$

where SOC(t) is the SOC at the current temperature, ${\mathrm{SOC}}_{T}\left(t-1\right)$ is the SOC converted from the previous moment to the current moment, and $C_{T}$ is the available capacity at different temperatures.

When the temperature changes from T1 to T2, the ${\mathrm{SOC}}_{T2}$ of the cell is:

(7) $${\mathrm{SOC}}_{T2}=\frac{{\mathrm{SOC}}_{T1}C_{T1}-{\mathrm{LFD}}_{T1-T2}}{C_{T2}}$$

When the temperature changes from T2 to T1, the ${\mathrm{SOC}}_{T1}$ of the cell is:

(8) $${\mathrm{SOC}}_{T1}=\frac{{\mathrm{SOC}}_{T2}C_{T2}+{\mathrm{LFD}}_{T1-T2}}{C_{T1}}$$

**Figure 9:**
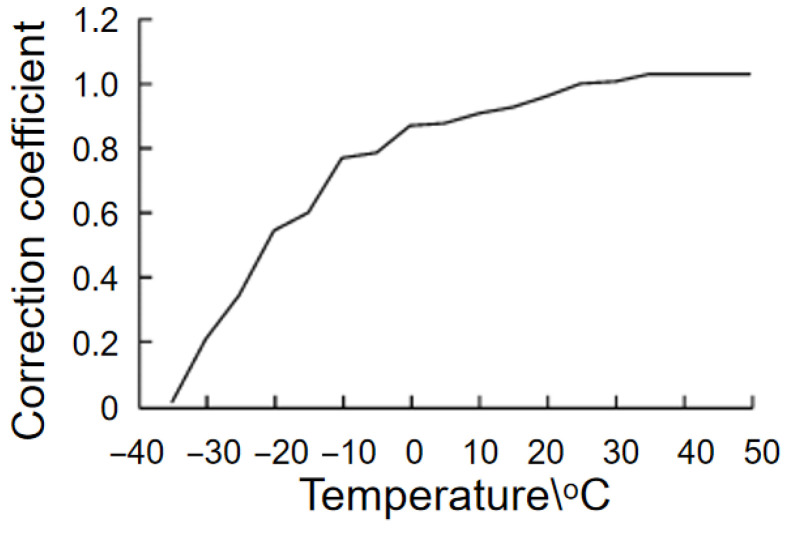
The temperature and temperature correction coefficient relationship.

Battery temperature will affect the actual capacity of the battery [7,28]. Figure 9 shows the temperature correction coefficient. It can be seen that when the temperature drops to −20 °C, the battery capacity is 50% of 25 °C; thus, battery temperature management is especially important, particularly in northern China, where cold battery temperature management is a key technology for application in electric cars.

The effect of the actual operating temperature on the actual capacity of the battery at 25 °C can be corrected as follows:

(9) $$C_{K}=\eta_{T}C_{b}$$

where Cb is the rated capacity of the battery at 25 °C, and $C_{K}$ is the actual capacity of the corrected battery at temperature K.

(3) Correction of aging factors

With the increase in the number of lithium-ion batteries, the battery will undergo an obvious aging phenomenon, and the capacity of the battery will be greatly reduced. If the traditional ampere-hour integral method is used to estimate the residual capacity, it will cause a large error, so the aging factor of the battery should be corrected. Both Refs. [23,29] conducted a large number of experiments to set the function of the aging coefficient and the cycle number of lithium-ion batteries as follows:

(10) $$\alpha =\left\{\begin{array}{c} 1,\;\;\;N\leq 500\\ 0.98,\;\;\;500<N<1000\\ 0.95,\;\;\;N\geq 1000 \end{array}\right.$$

**Figure 10:**
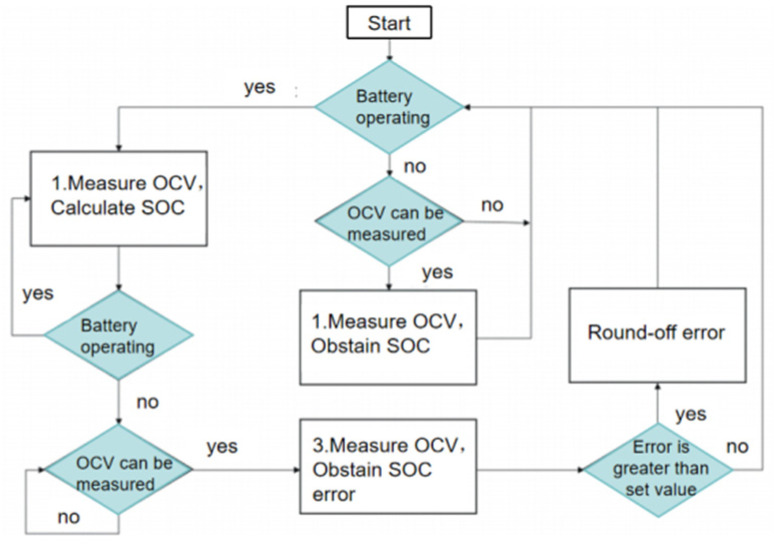
Flow chart of self-correction between SOC and OCV.

Due to the battery aging phenomenon, the relationship between OCV and SOC of the battery is not static, and the corresponding OCV–SOC of different aging stages is different, so the relationship between OCV and SOC should be corrected. When the difference between the calculated SOC and the actual SOC is greater than the set error critical value, the relationship between OCV and SOC is corrected. After the correction, the estimation of SOC will be more accurate, and the inaccuracy of residual capacity estimation caused by battery aging will be improved. The process of self-correction of the SOC and OCV relationship is shown in Figure 10 [30].

The battery aging rate will be affected by the charge and discharge rate, charge and discharge cut-off voltage, and temperature. Not considering these factors will cause error in the study of battery aging, so in order to effectively extend the service life of the battery, ensuring the safety of the battery, the charge and discharge rate and the charge and discharge depth should be reduced, and work should take place in the appropriate ambient temperature [31]. Through least-squares fitting, the relation between the capacity loss $C_{\mathrm{loss}}$ and the cycle times m of the battery aging can be obtained [32]:

(11) $$C_{\mathrm{loss}}=0.000006\;m^{2}+0.0026\;m+0.0255$$

(4) Other amendments

In addition to the above-mentioned initial battery power, temperature and aging factors will cause errors in the estimation of the ampere-hour integration method. The pertinence of SOC correction, the discharge impact factor and other factors will also affect the estimation of SOC, so these other factors should also be corrected.

For the situation of mining energy storage batteries often working at 90% SOC, the SOC of batteries in this stage should be corrected more specifically, and the corrected SOC is:

(12) $${\mathrm{SOC}}_{T}=\gamma \mathrm{SOC}+\left(1-\gamma \right){\mathrm{SOC}}_{0}$$

where γ is the correction factor, which is 0.82 [33].

When the battery is in the discharge state, it is discharged with different sizes, and the discharge capacity of the battery is different. When the battery is discharged with a small current, the discharge capacity is large. When the battery is discharged with a large current, the discharge capacity will gradually decrease. The relationship between the discharge influence factor I(η) and the current i is [34]:

(13) $$I\left(\eta \right)=1.937^{2}/\left(0.0488{\;*\;i}^{2}-0.3589\;*\;i+1.994\right)$$

In general, the ampere-hour integral method is simple, reliable and widely used. However, due to the accumulation of errors and the vulnerability to various factors, the improved ampere-hour integral method is often used for SOC estimation.

### 3.3. Electric Discharge Method

The discharge method involves using a constant current to discharge the battery. When the discharge is complete, the product of the current and the time is the discharge power. However, because the discharge method takes a lot of time and requires the working circuit to stop, it is not suitable for online estimation, so the discharge method is generally only used in the laboratory.

## 4. Estimation Method

The SOC estimation methods include the Kalman filter method (KF), the neural network method, and some other efficient estimation methods.

### 4.1. The Kalman Filter Algorithm

The Kalman filter (KF) algorithm uses the linear system equation of state to estimate the system state by observed input and output data. The basic principle is to adopt the state space model containing signal and noise, and update the estimate of the state variable with the state by using the estimated value of the previous moment and the observed value of the current moment. The Kalman filter algorithm includes the traditional Kalman filter algorithm, extended Kalman filter algorithm (EKF), trace Kalman filter algorithm (UKF) and so on. However, since real-world systems inherently exhibit nonlinearity, the KF algorithm is limited to linear problems. The extended Kalman filter (EKF) algorithm addresses this by applying a first-order Taylor series expansion to nonlinear functions. By omitting higher-order terms and retaining only the first-order components, EKF linearizes the state-space equations, enabling subsequent approximate system computation through the KF algorithm.

The state equation and the observation equation are shown in Equation (14) and Equation (15), respectively.

(14) $$\left\{\begin{array}{c} SOC_{k+1}=SOC_{k}-\frac{\eta TI_{k}}{Q}\\ U_{1,k+1}=U_{1,k}e^{\frac{-T}{\tau }}+R_{1}I_{1}\left(1-e^{\frac{-T}{\tau }}\right) \end{array}\right.$$

(15) $$U_{k}=U_{oc}-U_{1,k}-R_{o}I_{k}$$

Here, T denotes the sampling period, *U*_1_ is the voltage across resistor *R*_1_, η represents the charge–discharge efficiency, *I*_k_ is the load current at time k, and Q is the battery’s maximum available capacity.

The EKF algorithm does not depend on the initial value of SOC and has no accumulated error. The EKF algorithm was greatly influenced by the accuracy of the battery model and model parameters (resistance, electric capacity, etc.). The parameters of the battery change with many factors and exhibit nonlinearity and time variance. The average error of SOC estimated by the EKF algorithm is 1.91% under a 0.9 C constant current discharge and 2.35% under 1.3 C. The average error of SOC estimated by the EKF algorithm is 0.3%. The average error of SOC estimated by the EKF algorithm under the New European Driving Cycle (NEDC) is 0.2% [35]. The fuzzy–EKF–Ah model is used to fuse the two algorithms of EKF and integral Ah. The fused algorithm can effectively solve the problems of the cumulative error caused by the sampling accuracy of the Ah integral algorithm and the large estimation error of the EKF algorithm in the strong nonlinear interval [36].

The sampling point of interval time can affect the accuracy of SOC estimation. The battery parameters are received more accurately, and the SOC estimation error is smaller. When the sampling interval is 0.03 s, the SOC estimation error is the smallest. The second-order RC model can demonstrate the same result. When the sampling interval is 0.03 s, the SOC estimation error of the 1RC-1 method is less than 1.4%, the SOC estimation error of the 1RC-2 method is less than 0.017, and the root mean square error is 0.0087 and 0.01084. The conclusion is also used in the second-order RC model and contributes to the improvement in the SOC estimation accuracy [37].

For the established second-order RC equivalent model, the extended Kalman filter algorithm is used to estimate SOC, which can make the error between the actual SOC and the estimated value within 4% [38], improve the model of the lithium-ion battery, introduce the natural index function and increase the polynomial order, which can further improve the estimation accuracy of lithium-ion battery SOC based on the EKF algorithm [39].

The Kalman filter method can be used to estimate the SOC of a battery online, but it cannot observe the dynamic changes of the battery model. The double Kalman filter method can be used to estimate the battery status and parameters online, make the battery model have good adaptability, and improve the estimation accuracy of SOC. Experiments show that the maximum error of the algorithm is less than 4.5% [40]. A single estimation method may not adapt to all the SOC stages of the battery, so two or more methods can be combined to make the estimation error of the algorithm even smaller. The SOC value of the battery can be estimated by the ampere-hour integration method at 90–100%, and when the SOC value is below 90%, by the no-trace Kalman filter method. The overall error range of the combined algorithm is −1.5–1.0% [41].

The Kalman filter algorithm can effectively reduce the influence of noise and correct the initial power of SOC with high accuracy, but it also has high requirements on the system, the operation is complex, and it has a great dependence on the accuracy of the battery model.

### 4.2. Neural Network Method

The neural network method does not study the battery inside, but directly takes external factors such as battery voltage, current and temperature as input, and estimates the SOC based on the input data [42,43,44].

The accuracy of battery SOC estimation under dynamic conditions can be improved by combining the neural network method with the ampere-hour integral method, estimating battery charge–discharge efficiency with the cycle number and SOC value, estimating battery capacitance with temperature and average discharge current, and inputting these data into the ampere-hour integral method. Based on the BP neural network algorithm to estimate lithium-ion battery SOC, the collected battery data are implanted into the BP neural network model at the specified temperature for learning, training and verification. The results show that the error can be controlled to within 6% [45,46], which meets the requirements of SOC estimation and control.

The estimation error of the traditional BP neural network at low SOC values is bigger than with other conditions. A recurrent neural network estimation method is proposed. The parameters are important to the estimation accuracy of the neural network, and the maximum error of the recurrent neural network estimation method is limited to within 5% under two conditions [47]. A back-propagation neural network is established in multiple voltage segments. The nonlinear relationship between the decline in SOH and the change in the IC curve by the BP neural network is established, and the error is less than 2% [48].

The neural network method can make the estimation of SOC faster and more accurate, but it requires a large amount of sampling data and trains these data. It greatly increases the complexity of the system, so its application scope is not very wide. Further research should be carried out.

### 4.3. Other Methods

There are some other efficient SOC estimation methods, such as a proposed method based on the electrochemical impedance spectrum (EIS) that measures the EIS of a lithium-ion phosphate battery at different SOC and current values, summarizes the characteristics of battery EIS in terms of amplitude and phase, and establishes the battery SOC estimation algorithm based on the EIS [49]. The EIS can identify constants of LIBs [50,51]. The ohmic resistance and the real part of impedance at different frequencies exhibit correlations to SOC and SoH. The impedance is affected by relaxation time. However, the relaxation time is long-lasting for LIBs [50]. It is important to introduce the state of ionic diffusion [52,53,54]. Due to the different performance and characteristics of batteries in different stages, battery SOC is estimated in stages, and different estimation algorithms for different stages will greatly improve the estimation accuracy of battery SOC.

Deformation measurements emerge as an alternative for SOC estimation, especially in conditions where voltage-based methods are less effective [55]. A mechanical equivalent circuit model is introduced to simulate the battery deformation response to current profiles. Strain data are used to estimate the SoC and SoH of batteries, and SoC is estimated by the KF model and an artificial neural network [56]. SOC is estimated by current and terminal voltage with the associated measured force. The force-based SOC estimation algorithm extends the conventional SOC estimation approach [57].

Compared with the traditional method, the new SOC estimation method will greatly improve the estimation accuracy to a greater extent, but it also poses great challenges to the calculation amount of the system and the acquisition of sample data. Different estimation methods for different stages can make the estimation process of SOC more efficient.

## 5. Comparison of Different Estimation Methods

In summary, SOC estimation methods are endlessly emerging, and appropriate estimation methods should be selected on different occasions. The advantages and disadvantages of each estimation method are shown in Table 1.

Although there are many methods used to estimate battery SOC, each method has certain limitations. At present, the most widely used is the traditional SOC estimation method; that is, the ampere-hour integral method with the correction factor is used to estimate battery SOC, but the accumulation of error is inevitable. A single estimation method may not meet the estimation requirements. The accuracy of SOC estimated by the EKF algorithm depends on the accuracy of the battery parameters [53,54]. Batteries have nonlinear and time-varying characteristics. The battery performance model is based on the external characteristics, the model is built according to the voltage and current of the battery, and it is widely used. The network of resistors and capacitors (RCs) in series and in parallel can describe the external characteristics of the battery. Different battery parameters can be obtained at different SOCs. In the future, various algorithms can be integrated to foster strengths and avoid weaknesses, and different estimation methods can be adopted for different stages to minimize the possible error reduction and improve the accuracy of estimation.

## 6. Conclusions

In the application of electric vehicles, the use of batteries requires special care. Improper operations, such as overcurrent, overvoltage, overcharging, and overdischarging, can significantly accelerate the aging process of batteries, posing serious safety risks. There are many studies on the SOC of batteries, but most of them focus on methodology. Digital twin technology is used to estimate real-time monitoring, real-time feedback, and high-order zero-delay control of the battery stack and battery system, representing a future research trend. The energy distribution part of the battery system has significance for industrial production and energy storage.

As the most important parameter of the battery, the charge state of the battery needs to be accurately estimated. This paper summarizes the commonly used estimation methods for battery SOC—open-circuit voltage method, ampere-hour integral method, discharge method, Kalman filter method, and neural network method—introduces the influencing factors of the open-circuit voltage method and time integral method and their advantages and disadvantages, and finally summarizes various estimation methods and points out the comparison of the SOC estimation method in the future development direction.

## Figures and Tables

**Table 1:** Comparison of SOC estimation methods.

Estimation Method	Advantage	Shortcoming
Open-circuit voltage method	Simple and easy	The battery needs to be completely standing, with a lag effect; it is extremely difficult for the battery to remain in a fully open state.
Ampere-hour integration method	Simple and reliable, and widely used	There is a cumulative error, and it is greatly influenced by various factors; current measurement accuracy affects error.
Loss-of-charge method	Simple and convenient	It takes a lot of time and is not suitable for online estimation.
Kalman filter method	Dynamic estimation, with high accuracy	The system requirements are very high; parameter selection significantly affects the error.
Neural network method	Estimates accurately	Extensive sample data are needed.

## Data Availability

No new data were created or analyzed in this study. Data sharing is not applicable to this article.
